# Diagnostic Yield of Next-Generation Sequencing in Patients With Chronic Kidney Disease of Unknown Etiology

**DOI:** 10.3389/fgene.2019.01264

**Published:** 2019-12-13

**Authors:** Amber de Haan, Mark Eijgelsheim, Liffert Vogt, Nine V. A. M. Knoers, Martin H. de Borst

**Affiliations:** ^1^Department of Internal Medicine, Division of Nephrology, University Medical Center Groningen, University of Groningen, Groningen, Netherlands; ^2^Section Nephrology, Amsterdam Cardiovascular Sciences, Department of Internal Medicine, Amsterdam University Medical Centre, University of Amsterdam, Amsterdam, Netherlands; ^3^Department of Genetics, University Medical Center Groningen, University of Groningen, Groningen, Netherlands

**Keywords:** end-stage renal disease, exome sequencing, diagnostic utility, genetic testing, kidney disease

## Abstract

Advances in next-generation sequencing (NGS) techniques, including whole exome sequencing, have facilitated cost-effective sequencing of large regions of the genome, enabling the implementation of NGS in clinical practice. Chronic kidney disease (CKD) is a major contributor to global burden of disease and is associated with an increased risk of morbidity and mortality. CKD can be caused by a wide variety of primary renal disorders. In about one in five CKD patients, no primary renal disease diagnosis can be established. Moreover, recent studies indicate that the clinical diagnosis may be incorrect in a substantial number of patients. Both the absence of a diagnosis or an incorrect diagnosis can have therapeutic implications. Genetic testing might increase the diagnostic accuracy in patients with CKD, especially in patients with unknown etiology. The diagnostic utility of NGS has been shown mainly in pediatric CKD cohorts, while emerging data suggest that genetic testing can also be a valuable diagnostic tool in adults with CKD. In addition to its implications for unexplained CKD, NGS can contribute to the diagnostic process in kidney diseases with an atypical presentation, where it may lead to reclassification of the primary renal disease diagnosis. So far, only a few studies have reported on the diagnostic yield of NGS-based techniques in patients with unexplained CKD. Here, we will discuss the potential diagnostic role of gene panels and whole exome sequencing in pediatric and adult patients with unexplained and atypical CKD.

## Introduction

Chronic kidney disease (CKD) affects over 850 million individuals worldwide, and is associated with an increased risk of morbidity and mortality (Go et al., 2004; Gansevoort et al., 2013; Hill et al., 2016; Jager et al., 2019) and a high burden in terms of quality of life and costs (Smith et al., 2004; Baumeister et al., 2010; Eriksson et al., 2016; Nguyen et al., 2018). Recent predictions by the Institute of Health Metrics and Evaluations indicated that by 2040 CKD will be the fifth cause of life years lost on a global scale (Foreman et al., 2018).

CKD is defined as decreased renal function [an estimated glomerular filtration rate (eGFR) lower than 60 ml/min/1.73 m^2^] or the presence of kidney damage, for 3 months or longer, irrespective of the underlying cause (Levey et al., 2011). According to the Kidney Disease Improving Global Outcomes (KDIGO) guidelines, CKD can be classified into six stages based on eGFR (**Table 1**) and into three categories based on albuminuria (**Table 2**) (KDIGO, 2013). The first stages of CKD are often clinically silent and most patients start developing symptoms in stages 4 and 5, hampering the early diagnosis of CKD.

**Table 1:** Estimated glomerular filtration rate stages for chronic kidney disease.

GFR stage	eGFR	Description
**1**	>90	Normal or high
**2**	60–89	Mildly decreased
**3a**	45–59	Mildly to moderately decreased
**3b**	30–44	Moderately to severely decreased
**4**	15–29	Severely decreased
**5**	<15	Kidney failure

GFR, glomerular filtration rate; eGFR, estimated glomerular filtration rate. Based on KDIGO guidelines (KDIGO, 2013).

**Table 2:** Albuminuria categories for chronic kidney disease.

Albuminuria category	ARC range		Description
	mg/g	mmol/g	
**1**	<30	<3	Normally to mildly increased
**2**	30–300	3–30	Moderately increased
**3**	>300	>30	Severely increased

ACR, albumin-to-creatinine ratio. Based on KDIGO guidelines (KDIGO, 2013).

A positive family history is reported by 24–34% of patients with CKD, and familial clustering is a common phenomenon in patients with end-stage renal disease (ESRD) (Akrawi et al., 2014; Skrunes et al., 2014; Connaughton et al., 2015). This indicates that, during the diagnostic workup of a CKD patient, the possibility of a hereditary cause cannot be neglected. This is supported by the fact that monogenic mutations are frequently found in early-onset CKD and that inherited kidney diseases are a common cause of ESRD in both adults and children (Devuyst et al., 2014).

Approximately 17% of patients with ESRD do not have a primary renal disease (PRD) diagnosis, and are therefore labeled as CKD with unknown origin. Furthermore, in many patients the primary diagnosis is inaccurate (Hoekstra et al., 2017). Making a correct diagnosis in these patients may have therapeutic implications. For example, a genetic mutation predisposing to atypical hemolytic uremic syndrome (aHUS) may provide crucial therapeutic possibilities with the availability of specific monoclonal antibodies targeting the complement system (Zuber et al., 2012). Moreover, a genetic diagnosis may be of pivotal importance for family counseling and in the setting of kidney transplantation, particularly when living-related donation is involved (Aymé et al., 2017).

A recent study in a cohort of CKD patients, of whom 65% had ESRD, demonstrated that a molecular diagnosis can be derived with next-generation sequencing (NGS) in approximately one in ten adults (Groopman et al., 2018). Since unexplained diseases have a higher risk of a genetic origin, it is likely that the proportion of patients in whom a molecular diagnosis can be identified is even larger when considering patients with unexplained CKD, or those with an atypical presentation. However, genetic testing is currently only performed in a minority of these patients.

NGS-based techniques are thus likely to improve the diagnostic accuracy in patients with CKD of unknown origin. It could also improve patient care, since NGS-based testing has the potential to identify the cause of CKD in an early stage of the disease, which allows timely intervention to delay or prevent development of ESRD. However, given the novelty of the technique, the value of NGS-based multi-gene panels and whole exome sequencing for these patients in clinical practice remains to be demonstrated.

In this review, we will discuss the utility of NGS-based testing in CKD patients with childhood-onset and adult-onset disease. Subsequently, we will focus on the potential role of NGS in the diagnostic workup of adult patients with an unknown PRD.

## Prevalence and Causes of Chronic Kidney Disease

The global prevalence of CKD is 13.4%, with a higher prevalence in women compared to men and in patients over 70 years of age (Mills et al., 2015; Hill et al., 2016). Data on trends in the prevalence of CKD over the past decades are conflicting (Coresh et al., 2007; Aitken et al., 2014; Murphy et al., 2016), and the prevalence of CKD varies widely across countries (Bruck et al., 2016; de Grauw et al., 2018). For example, in Europe the prevalence estimates for CKD stage 1–5 vary from 3.3% in Norway, to 10.4% in the Netherlands, and 17.3% in north-eastern Germany (Bruck et al., 2016; de Grauw et al., 2018). CKD prevalence may even vary within one country (Zhang et al., 2012). There are many factors which can partially explain the reported differences in prevalence between and within countries. Healthcare organization and policies in prevention and screening programs for CKD and its risk factors may vary substantially between countries. Other factors include, but are not limited to, communicable diseases, variations in laboratory methods, environmental factors like diet and toxins, ethnicity, genetic susceptibility, and differences in the prevalence of CKD risk factors like diabetes, hypertension and obesity (Jha et al., 2013; Agyemang et al., 2016; Stanifer et al., 2016; Glassock et al., 2017; Webster et al., 2017).

While hypertension and diabetes are the leading CKD causes in Western countries, other common causes include glomerulonephritis and inherited forms of CKD (Jha et al., 2013; Mallett et al., 2014). Interestingly, hypertension is not only a cause of CKD, but it can also occur as a result of CKD. Therefore, it can sometimes be difficult to determine which occurred first (Hamrahian and Falkner, 2017). It could thus be possible that some CKD patients with the PRD diagnosis "hypertensive nephropathy" have a different disease etiology, with hypertension being rather a consequence than the primary cause of the disease. In clinical practice, isolated essential hypertension rarely leads to ESRD, in contrast with, for example, malignant hypertension.

A hereditary form of CKD can be found in approximately 10% of the adult CKD population, comprising both patients with ESRD and patients with pre-dialysis kidney disease (Devuyst et al., 2014; Mallett et al., 2014). Compared to adults, children with CKD are more likely to have a hereditary form of CKD. Current estimates are that at least 15–20% of early-onset CKD, that is before 25 years of age, is caused by a genetic form. It is also estimated that nearly all children who progress to ESRD have an inherited form of CKD (Devuyst et al., 2014; Vivante and Hildebrandt, 2016). The most common inherited kidney diseases seen in the pediatric and adult populations are likely to be different from each other. For instance, autosomal dominant polycystic kidney disease (ADPKD) usually presents in adulthood, while nephronophthisis is more prevalent in children (Hildebrandt, 2010).

The number of ESRD patients with an unknown PRD diagnosis is increasing. In the Netherlands, the prevalence of ESRD of unknown etiology is 17% (Hoekstra et al., 2017). Similar percentages are found elsewhere in Europe (Kramer et al., 2018). Patients with an unknown PRD would benefit from an earlier diagnosis, which could permit early intervention to reduce the risk of cardiovascular complications and slow down or prevent the progression of CKD.

## Next Generation Sequencing

Genetic testing can have an important role in accurately diagnosing PRD in cases that have a higher risk of an inherited form of CKD; for example patients with familial CKD, early-onset CKD, an unusual disease course, or patients with an unknown cause of CKD (Vivante and Hildebrandt, 2016). One of the benefits of genetic testing is that it can identify the cause of disease regardless of disease stage, contrary to diagnostic renal biopsies that often fail to reveal a diagnosis in very early or late stages of the disease (Renkema et al., 2014; Stokman et al., 2016). Moreover, genetic testing is non-invasive and can shorten the diagnostic odyssey. Identification of a genetic diagnosis can play an important role in family planning, options for kidney transplantation, targeted surveillance of extra-renal features and can guide treatment and genetic counseling (Adams and Eng, 2018; Nestor et al., 2018; Armstrong and Thomas, 2019). Counseling and genetic testing can also be of importance for relatives of the patient with genetic CKD. Identification of a genetic risk variant in otherwise healthy relatives can make them eligible for monitoring and early intervention to prevent progression of CKD and its complications, such as cardiovascular disease. In addition, it can exclude the relative for living kidney donation as to reduce the risk of future CKD.

Diagnostic genetic testing has benefited from advances in NGS, which has enabled cost-effective sequencing of large regions of the genome (Bick and Dimmock, 2011; Petersen et al., 2017). NGS, or massively parallel sequencing, allows simultaneous sequencing of multiple genes associated with a particular phenotype (gene panels), all 20,000 protein-coding genes (whole exome-sequencing), or the entire genome (whole genome sequencing) (Adams and Eng, 2018; Groopman et al., 2018). Each approach has its own advantages and limitations. Gene panels often have a higher sequencing coverage and depth than whole exome sequencing (WES) or whole genome sequencing (WGS), resulting in a greater diagnostic yield (Xue et al., 2015). However, the coverage and depth of NGS-based techniques varies depending on the capture systems used (Chilamakuri et al., 2014; Shigemizu et al., 2015; García-García et al., 2016). Coverage and depth of a system will also change over time as new updates and techniques become available, such as capture free WGS. It is thus important to select a suitable system.

Gene panel testing reduces the risk of incidental findings, being designed to only include a pre-selected subset of genes. Disadvantages of gene panels are that they need to be regularly updated to include newly discovered genes and the inherent requirement of a correctly interpreted clinical context, in order to avoid an inappropriate gene panel to be performed (Prakash and Gharavi, 2015; Groopman et al., 2018).

In contrast with gene panels, WES sequences all the protein-coding genes of the genome and is thus not limited to a preselected set of genes. This allows a more flexible analysis compared to gene panels and the opportunity to identify new causative genes. Furthermore, WES data can be stored for future reanalysis as new genes are discovered and variants are reclassified (Groopman et al., 2018; Jayasinghe et al., 2018). Although WES sequences the entire exome, it is possible to only target a specified subset of genes with an *in silico* panel (targeted WES). This approach gives similar results as gene panels, but has the advantage that the original WES data can be “opened” for further analysis if new genes are discovered or if a causative variant cannot be identified in the initial analysis (Preston et al., 2017; Jayasinghe et al., 2018). Because of these advantages, most of the current diagnostic gene panels are based on targeted WES.

An important issue when performing WES is the possibility of detecting incidental findings, which are causative variants not related to the primary purpose for genetic testing. For example, when a genetic variant that predisposes to cancer is identified in a patient undergoing WES for hereditary kidney disease, this finding could not only have consequences for the patient (for instance intensified cancer screening or preventive surgery), but also for the relatives of the patient. The identification of a genetic risk variant for cancer can also influence treatment. Kidney transplant recipients have an increased risk of developing cancer, which can be partly attributed to immunosuppressive therapy. To minimize the risk of developing cancer, reduction of immunosuppression or other treatment options should be considered in transplant recipients with a genetic predisposition for cancer. There is, however, no consensus about when to report an incidental finding.

Another drawback of WES is that not all genomic regions are equally covered and regions with high guanine-cytosine (GC) rich content, copy number variants, and high sequence homology with pseudogenes may be missed (Xue et al., 2015). For example, WES is of limited use in diagnosing ADPKD, which is caused by mutations in *PKD1* and *PKD2*. The *PKD1* gene has a high degree of sequence homology with six pseudogenes, which complicates variant identification (Ali et al., 2019).

Some of the limitations of WES can be addressed by WGS. For instance, WGS can identify copy number variants and has a more complete per base coverage compared to WES (Belkadi et al., 2015; Wu et al., 2016). WGS has the same advantages as WES, but has the additional benefit of being able to sequencing nearly all types of genetic variations in both the coding and non-coding regions of the genome (Taylor et al., 2015; Wu et al., 2016; Lionel et al., 2018). For instance, WGS was able to identify a genetic variant in 86% of patients with ADPKD. This suggest that WGS can discriminate between the original *PKD1* gene and the pseudogenes (Mallawaarachchi et al., 2016).

Other studies have reported that the diagnostic yield of WGS is higher than WES in a variety of disorders and that WGS can identify a causative variant in 20–40% of the patients in whom no genetic cause could be identified with WES (Gilissen et al., 2014; Ellingford et al., 2016). Nevertheless, WGS is not commonly used in clinical practice. This could be due to the costs and time associated with WGS, the requirements for data analysis and data storage, and the complex interpretation of unknown variants, especially intronic and other non-coding variants. However, it is likely that the capability to interpret variants in noncoding regions of the genome will improve over time. This, together with an expected decline in sequencing costs, will increase the advantages of WGS in the future (Lionel et al., 2018).

It is important to recognize that all forms of NGS-based testing have common limitations. For example, all NGS-based techniques were unable to detect causative variants in *MUC1* in six unrelated families with autosomal dominant tubulointerstitial kidney disease. It was likely missed due to the highly repetitive GC-rich sequence and the variant was only identified by long-range polymerase chain reaction and molecular cloning (Kirby et al., 2013). NGS-based testing is furthermore of limited value in most patients with acquired diseases and the translation of genetic findings to clinical practice may be challenging (Stokman et al., 2016). In addition, the social, ethical, and legal concerns of genetic testing cannot be neglected (Guay-Woodford and Knoers, 2009; Clarke, 2014).

Some limitations of NGS are caused by the short-read lengths that are used to maintain high coverage and depth. A new technique, long-read sequencing, may overcome some NGS-specific limitations. Long-read single molecule platforms offer the ability to directly evaluate many difficult or even previously unsequenceable regions of the genome, such as repetitive elements, non-targeted structural variant breakpoints at base-pair resolution, pseudogene discrimination, and epigenetics (Mantere et al., 2019). In the future, long-read sequencing based WGS could thus increase the diagnostic yield in patients with genetic CKD and give more patients a (correct) PRD diagnosis. Because of the advantages of genetic testing in general, and specifically the advent of NGS in the past decade, genetic testing is becoming increasingly relevant in clinical practice. However, the interpretation of variants remains a challenge with both WES and WGS.

Most studies on genetic testing in the field of nephrology are restricted to a research setting (e.g., cohort studies), and so far little is known about its diagnostic utility in clinical practice.

## Next-Generation Sequencing in Children With Chronic Kidney Disease

The most common causes of renal disease in children are congenital anomalies of the kidney and urinary tract (CAKUT), glomerulonephritis, steroid-resistant nephrotic syndrome (SRNS) and renal ciliopathies (Harambat et al., 2012; Becherucci et al., 2016). These renal diseases can all have a genetic etiology. Reports show that in approximately 20% of adolescents with early-onset CKD a monogenic cause of disease can be identified (Devuyst et al., 2014; Vivante and Hildebrandt, 2016). The high contribution of genetic causes in pediatric CKD is underlined by the fact that almost all children who progress to ESRD have an inherited form of CKD.

Extensive genetic research has been done in the pediatric CKD population, although data obtained in the clinical practice setting remain scarce. For example, a recent study investigated the diagnostic yield of targeted WES in a pediatric kidney transplant recipient cohort. The authors included children irrespective of clinical PRD diagnosis, and identified a causative variant in 34 of 104 (32.7%) of patients (Mann et al., 2019). They found a higher diagnostic yield in patients with a positive family history for CKD, patients with extra-renal manifestation or with a history of consanguinity (Mann et al., 2019).

Other studies examined the diagnostic yield of NGS-based testing in pediatrics cohorts with a specific phenotype. For example, with NGS a genetic cause was identified in 55–80% of patients with Alport syndrome and in 21–25% of patients with nephronophthisis-related ciliopathies (Fallerini et al., 2014; Moriniere et al., 2014; Schueler et al., 2016). In patients with CAKUT, gene panels identified a causative variant in 2.5–6% (Hwang et al., 2014; Kohl et al., 2014), whereas targeted WES identified diagnostic variants in 5–14% of patients with CAKUT (Bekheirnia et al., 2017; van der Ven et al., 2018).

The diagnostic utility of targeted WES has also been addressed in SRNS, where a diagnostic variant was found in 11.1–29.5% of patients (Sadowski et al., 2015; Bierzynska et al., 2017; Tan et al., 2018; Warejko et al., 2018).

Gene panels identified a causative variant in 16.8–20.8% of patients with childhood onset nephrolithiasis, which is significantly higher than the adult-onset setting where a genetic cause was identified in 11.4% (Halbritter et al., 2015; Braun et al., 2016). In comparison, targeted WES in patients with nephrolithiasis with onset before the age of 25 years identified a diagnostic variant in 29.4%. According to the authors, the higher diagnostic yield can be attributed to a younger age of onset, the inclusion or more familial cases, and presence of consanguinity (Daga et al., 2018).

The nephrolithiasis studies show that the clinical characteristics of patients can influence the diagnostic yield. The study design (sample size, number of genes sequenced, and whether patients are recruited from tertiary hospitals or specialized centers) can likewise affect the diagnostic yield. In addition, most studies have been conducted in patients predominantly of European ancestry and the result may therefore not always apply to other ethnic groups. The results from the mentioned studies can therefore not always be generalized to the broader CKD population.

The data from the abovementioned studies are mostly obtained in a research setting. However, the potential diagnostic utility of targeted WES has also been shown in a clinical practice setting. Offering targeted WES to families of children with familial CKD led to an overall diagnostic yield as high as 46% (Mallett et al., 2017). These findings highlight the diagnostic potential of NGS-based testing in pediatric patients with early-onset CKD. It also underlines the need for further research in a diagnostic setting, to further define the position of NGS-based diagnostics in clinical practice.

## Next-Generation Sequencing in Adults With Chronic Kidney Disease

Genome-wide association studies (GWAS) and GWAS meta-analyses have identified several common variants in genetic loci associated with CKD, including variants in *UMOD*, *SHROOM3*, solute carriers, and E3 ubiquitin ligases, as reviewed elsewhere (Cañadas-Garre et al., 2019). Despite the common genetic risk variants and the relatively high prevalence of genetic causes of CKD in adults, the role of NGS diagnostics in clinical practice has been limited compared to the pediatric population. A possible explanation is that genetic testing has traditionally been considered to be more successful in children compared to adults (Mallett et al., 2017). However, targeted WES in familial CKD in a diagnostic setting showed similar diagnostic rates among children (31 of 67 patients, 46.3%) and adults (27 of 68 patients, 39.7%) (Mallett et al., 2017). There were, however, significant differences in diagnostic yield between adults and children for certain phenotypes such as aHUS (17% in adults *vs.* 67% in children) (Mallett et al., 2017).

These findings were confirmed by a recent study from Ireland. Targeted WES showed comparable diagnostic rates among pediatric onset CKD (20 of 50 patients, 40%) and adult onset CKD (35 of 85 patients, 41%) (Connaughton et al., 2019). The similar overall diagnostic rate between adults and children seems in contrast with previous observations that showed an inverse correlation between proband age and the chance of identifying a genetic cause for a specific inherited type of kidney disease (Sadowski et al., 2015).

Subsequent studies have further highlighted the diagnostic potential of NGS in an adult CKD population. For example, targeted WES in a broad CKD population identified diagnostic variants in 307 of 3,315 (9.3%) patients (Groopman et al., 2018). This cohort consisted of 91.6% adults (over 21 years of age) and 64.7% of the patients had ESRD. In the 307 patients with a genetic diagnosis a subsequent analysis was done with a phenotype-specific gene panel. This resulted, at most, in a genetic diagnosis in 136 of 307 (44.3%) patients (Groopman et al., 2018).

Several studies have investigated the diagnostic rate of NGS-based testing in adults with specific renal phenotypes. For example, genetic testing identified causative variants in 14% of adult-onset SRNS and gene panels found diagnostic variants in 11.4% of adult-onset nephrolithiasis. In both studies a lower genetic yield was associated with a higher proband age (Santín et al., 2011; Halbritter et al., 2015).

The diagnostic yield of NGS-based testing in patients with no clear PRD or with a family history of CKD is likely higher than in the overall CKD population. Indeed, a pilot study in adults with familial or unexplained CKD demonstrated that targeted WES identified a causative variant in 22 of 92 (24%) patients. These encompassed 13 different genetic disorders and influenced medical management in 21 of 22 patients (Lata et al., 2018).

As mentioned before, the results from these studies cannot always be generalized to the broader CKD population due to ascertainment bias. For instance, the authors of the pilot study mention that the generalizability of the results to the general CKD population is limited by a small sample size and the fact that the cohort is enriched for familial or suspected genetic CKD (Lata et al., 2018).

 These studies confirm the importance of NGS-based testing in adults with CKD. Increased awareness for the possibility of genetic testing in adults could help to provide more patients an early and specific diagnosis, aiming to prevent progression of CKD and its complications. However, the diagnostic yield of genetic testing may be lower in patients who are older at disease onset. More research is needed to determine the effect of age at onset on the yield of genetic testing in the context of CKD; such studies will direct guidelines towards cost-effective implementation of NGS-based testing. Moreover, the diagnostic yield in CKD is currently limited by the fact that in many cases the underlying cause is polygenic in nature; the development of polygenic risk scores may further improve diagnostic yield in the future.

## Next-Generation Sequencing to Reclassify the Primary Renal Diagnosis

Most inherited kidney diseases have a high degree of genetic heterogeneity and are associated with a wide range of phenotypes (Kalatharan et al., 2018). This can make it challenging to distinguish certain kidney diseases based on clinical presentation, and may lead to misdiagnosis.

The emergence of NGS has enabled a more precise differentiation between overlapping phenotypes based on a genetic diagnosis. In some cases, the genetic diagnosis can lead to reclassification of the PRD diagnosis in individual patients. For example, it may be difficult to discriminate between Alport syndrome, mesangial proliferative glomerulonephritis (MPGN), and primary focal segmental glomerulosclerosis (FSGS) based on clinical presentation and renal biopsies (Yao et al., 2012). However, the distinction between these diseases is of great importance with respect to treatment and clinical outcomes. Genetic studies have shown that patients with Alport syndrome have been misdiagnoses as MPGN (Adam et al., 2014). In addition, multiple studies have shown that patients with FSGS were reclassified as Alport syndrome based on mutations found in *COL4A3*, *COL4A4*, and *COL4A5* (Adam et al., 2014; Gast et al., 2016; Yao et al., 2019). Genetic testing can distinguish between different etiologies in heterogeneous diseases and prevent an incorrect diagnosis.

Another example of reclassification has been illustrated in a report describing the use of WES to obtain a genetic diagnosis (Choi et al., 2009). In a cohort of patients with suspected Bartter syndrome, WES identified no candidate variants in Bartter-associated genes but instead found a variation in *SLC26A3*. Variants in this gene are associated with chloride diarrhea, and clinical follow-up confirmed the diagnosis of chloride diarrhea in these patients (Choi et al., 2009).

Moreover, in a recently published analysis from the iGeneTrain consortium, it was found that 0.5% of patients with adult-onset ESRD had a full gene deletion of the *NPHP1* gene. This percentage increased to almost 1% when only the patients of 18–50 years at ESRD onset were considered (n = 24). Full gene deletions of *NPHP1* are associated with nephronophthisis, the most common genetic cause for ESRD in children. Of the 26 patients with a full *NPHP1* gene deletion, only 3 had a correct clinical PRD diagnosis of nephronophthisis (Snoek et al., 2018). This is not surprising, since nephronophthisis had previously been considered to result in ESRD exclusively at child age.

Other studies have also reported on the reclassification of PRD diagnosis based on genetic findings, albeit in cohorts with limited sample size. For example, Lata et al. identified a genetic diagnosis in 22 of 92 adults with CKD, and in two of these patients the kidney disease was reclassified. The reclassification of diagnosis, from FSGS to autosomal dominant Alport syndrome and to autosomal recessive Alport syndrome, had direct consequence for clinical management (Lata et al., 2018). Furthermore, in a study by Connaughton et al. targeted WES identified a molecular diagnosis in 42 of 114 families with CKD, and in 9 of these 42 families the initial CKD diagnosis was changed based on genetic findings (Connaughton et al., 2019).

Similarly, in a pediatric cohort consisting of 79 consanguineous or familial cases with a suspected diagnosis of nephronophthisis, targeted WES identified causal variants in 63% of cases. While in 64% of these families the suspected diagnosis was confirmed, in a noteworthy 36% of these families a different molecular diagnosis was found. The CKD diagnoses in these families were reclassified as Alport syndrome (8%), CAKUT (6%), renal tubulopathies (16%), autosomal-recessive polycystic kidney disease (4%), and auto-immune nephropathy (2%) (Braun et al., 2016). These finding show that (targeted) WES can provide a correct diagnosis in genetically and phenotypically heterogeneous diseases. The above findings demonstrate that it is very likely that a subset of CKD patients has a misdiagnosis and that with the increased availability of genetic testing more primary diagnoses will be reclassified.

## Next-Generation Sequencing in Unexplained Chronic Kidney Disease

WES facilitated the molecular diagnosis and PRD reclassification in both pediatric and adult cohorts and informed prognosis, therapy, and counseling in these patients. Moreover, WES has the potential to resolve cases with unknown etiology (Need et al., 2012; Hauer et al., 2018) and could thus be a valuable analytical tool in the diagnostic process of patients with an unknown PRD or in patients who have an atypical/nonspecific clinical presentation.

The diagnostic value of WES in CKD patients with unknown etiology is illustrated by a pilot study by Lata et al, including 16 patients who had CKD with unknown etiology (Lata et al., 2018). A causative variant was identified in nine of these patients, indicating a diagnostic yield of 56%. The established diagnoses consisted of a variety of nephropathies, including CHARGE syndrome, X-linked and autosomal forms of Alport syndrome, *HNF1B*-associated disease, Dent’s disease, and autosomal dominant tubulointerstitial kidney disease (**Table 3**). In addition to providing a kidney disease diagnosis for these patients, the genetic diagnosis influenced medical management. Clinical implications included a change in therapy (for example avoidance of immunosuppressive therapy), screening of at-risk family members, screening for extra-renal features, and optimal selection of living related kidney donors (Lata et al., 2018).

**Table 3:** Overview of studies on the diagnostic yield of next-generation sequencing-based testing in patients with unexplained chronic kidney disease.

	N sequenced, unexplained/total cohort	Age at presentation	Patients characteristics	Positive family history, n (%)	New (genetic) diagnoses	Number of genes in NGS panel	Yield in unexplained CKD, %
(Lata et al., 2018)	16/92	37 (SD 11) years	Recruited from tertiary care medical center, predominantly Caucasian	9 (56%)	CHARGE syndrome, DENT’s disease, AS, *HNF1B* associated disease, ADTKD, PARN mutations (n = 3)	287	56
(Groopman et al., 2018)	281/3315	At inclusion 91.6% of patients is older than 21 years*	Recruited from the AURORA study (Fellström et al., 2009) and a tertiary care medical center, more than 60% has ESRD, 35.6% non-European ancestry**	Available for 2187 patients: 619 (28.3%)†	NA	625	17.1
(Connaughton et al., 2019)	34/114 families	36% childhood onset, 62% adult onset*	Recruited from nephrology services in Ireland, predominantly Caucasian, included mostly patients with positive family history or extra-renal features, 65% has ESRD**	33 (80%)	Cystic kidney disease or NPHP, syndromic CAKUT, AS, tubulointerstitial kidney disease, hypertensive nephropathy, nephrocalcinosis/nephrolithiasis	478	47
(Ottlewski et al., 2019)	50/135	Median age at ESRD 43.3 (range 15.4–66.5)	Recruited from waitlist for kidney transplantation at a tertiary care medical center, predominantly Caucasian	NA	AS, familial FSGS	209	12

ADTKD, autosomal dominant tubulointerstitial kidney disease; AS, Alport syndrome; CHARGE, coloboma, heart defects, atresia choanae, growth retardation, genital abnormalities and ear abnormalities; CKD, chronic kidney disease; ESRD, end-stage renal disease; FSGS, focal segmental glomerulosclerosis; NA, not available; NPHP, nephronophthisis; SD, standard deviation.

*Age at presentation from the total cohort, not specific for unexplained CKD subset.

**Patient characteristics from the total cohort, not specified for unexplained CKD subset.

^†^Number of patients with a positive family history for CKD in the total cohort, not specified for unexplained CKD subset.

Another study in which the diagnostic value of WES in patients with undiagnosed CKD is demonstrated is the recently published study of Groopman et al. Targeted WES provided a molecular diagnosis in 48 of 281 (17.1%) patients with CKD of unknown origin. The diagnostic yield in this group was the second highest in this study, the yield was only higher in patients with a clinical diagnosis of congenital or cystic renal disease (23.9%) (**Table 3**) (Groopman et al., 2018). In addition, the authors found that nephropathy of unknown origin, a family history of CKD, and clinical diagnosis of congenital or cystic renal disease were independent predictors of yielding a genetic diagnosis (Groopman et al., 2018).

A recent study by Connaughton et al. also reported on the diagnostic value of targeted WES in patients with unexplained CKD (Connaughton et al., 2019). A causative variant was identified in 16 of 34 (47%) families with CKD of unknown origin. The newfound diagnoses consisted, among others, of X-linked and autosomal forms of Alport syndrome, Dent’s disease, Fanconi anemia, Wolfram-like syndrome, nephronophthisis, and nephrocalcinosis/nephrolithiasis. The diagnostic yield in the unexplained CKD family group represented 38% of the identified molecular diagnosis in this study (16 of 42 families) (Connaughton et al., 2019) (**Table 3**).

The diagnostic value of gene panels in patients with undetermined ESRD is demonstrated by Ottlewksi *et al*, who tested 50 patients on the kidney transplantation waiting list with undetermined CKD (Ottlewski et al., 2019). The renal gene panel, consisting of 209 genes associated with ESRD, identified a causative variant in 6 of 50 (12%) these patients. The genetic diagnosis consisted of X-linked and autosomal dominant forms of Alport syndrome and familial FSGS. The identification of a genetic diagnosis in six patients significantly reduced the proportion of patients with unexplained ESRD on the kidney transplant waiting list (Ottlewski et al., 2019) (**Table 3**).

The dissimilarities in diagnostic yield between these studies likely result from differences in sample size, different inclusion criteria, NGS approach, and different selection of genes. The study of Lata et al. included only patients with familial or suspected genetic CKD (Lata et al., 2018). This enrichment for genetic diagnosis could explain, together with the limited sample size, the high yield. Groopman et al. included CKD patients irrespective of diagnosis or family history (Groopman et al., 2018), while Connaughton et al. included mostly CKD patients with a positive family history or extra-renal features and only a small proportion of CKD patients who had neither familial CKD nor extra-renal features (Connaughton et al., 2019). The study by Ottlewski et al. also has a limited sample size, but it is not clear what the proportion of patients with familial CKD or extra-renal features is. However, five of six patients in which a genetic diagnosis was identified had a positive family history (Ottlewski et al., 2019). In addition, it is the only study using gene panels instead of targeted WES. The diagnostic yield is thus dependent on the characteristics of the included patients and the study design. This can make it difficult to make comparisons between different studies, especially when some patient characteristic are unknown.

The findings above highlight the diagnostic value of NGS based testing for patients with unexplained CKD, especially in patients with early-onset or familial CKD. Further research is needed to explore the diagnostic utility of WES for CKD of unknown etiology in larger cohorts and in a clinical setting. Such studies should guide the position of NGS diagnostics in the work-up of patients with unknown PRD, by revealing subpopulations with the highest diagnostic yield that may be preferentially offered this diagnostic testing.

## Conclusion

NGS, including gene panels and WES, shows promising results as a diagnostic tool in both pediatric and adult CKD cohorts. In a considerable proportion of patients, particularly in those with familial CKD (∼40%), NGS has the potential to resolve CKD cases with an unknown etiology. NGS also enables the reclassification of PRD diagnoses. Counseling on the potential implications of a positive test result is essential, particularly for WES, given the possibility of incidental findings. Although further research is needed to determine which subpopulations will have the highest diagnostic yield, we foresee an expanding role for NGS-based diagnostics in clinical nephrology in the coming years.

## Author Contributions

AH wrote the first draft of the manuscript. ME, LV, NK, and MB gave feedback and contributed to manuscript revision. All authors read and approved the submitted version.

## Funding

This work has been supported by a public-private collaboration between the University Medical Center Groningen, The Netherlands, and Sanofi Genzyme. This collaboration project is co-financed by the Ministry of Economic Affairs and Climate Policy by means of the PPP Allowance made available by the Top Sector Life Sciences & Health to stimulate public-private partnerships.

## Conflict of Interest

MB has consultancy agreements with Amgen, Astra Zeneca, Bayer, Vifor Fresenius Medical Care Renal Pharma, and Sanofi Genzyme, and received grant support from Amgen and Sanofi Genzyme.

The remaining authors declare that the research was conducted in the absence of any commercial or financial relationships that could be construed as a potential conflict of interest.
